# Hemozoin Induces Hepatic Inflammation in Mice and Is Differentially Associated with Liver Pathology Depending on the *Plasmodium* Strain

**DOI:** 10.1371/journal.pone.0113519

**Published:** 2014-11-24

**Authors:** Katrien Deroost, Natacha Lays, Thao-Thy Pham, Denisa Baci, Kathleen Van den Eynde, Mina Komuta, Mauro Prato, Tania Roskams, Evelin Schwarzer, Ghislain Opdenakker, Philippe E. Van den Steen

**Affiliations:** 1 Department of Microbiology & Immunology, Laboratory of Immunobiology, Rega Institute for Medical Research, KU Leuven - University of Leuven, Leuven, Belgium; 2 Department of Oncology, University of Torino, Torino, Italy; 3 Department of Biology, Tor Vergata Rome University, Rome, Italy; 4 Translational Cell & Tissue Research, KU Leuven – University of Leuven, Leuven, Belgium; 5 Department of Neuroscience, University of Torino, Torino, Italy; 6 Department of Genetics, Biology, and Biochemistry, University of Torino, Torino, Italy; Museum National d'Histoire Naturelle, France

## Abstract

Malaria is a global disease that clinically affects more than two hundred million people annually. Despite the availability of effective antimalarials, mortality rates associated with severe complications are high. Hepatopathy is frequently observed in patients with severe malarial disease and its pathogenesis is poorly understood. Previously, we observed high amounts of hemozoin or malaria pigment in livers from infected mice. In this study, we investigated whether hemozoin is associated with liver injury in different mouse malaria models. C57BL/6J mice infected with the rodent parasites *Plasmodium berghei* ANKA, *P. berghei* NK65 or *P. chabaudi* AS had elevated serum liver enzymes without severe histological changes in the liver, in line with the observations in most patients. Furthermore, liver enzymes were significantly higher in serum of *P. chabaudi* AS-infected mice compared to mice infected with the *P. berghei* parasite strains and a strong positive correlation was found between hepatic hemozoin levels, hepatocyte damage and inflammation in the liver with *P. chabaudi* AS. The observed liver injury was only marginally influenced by the genetic background of the host, since similar serum liver enzyme levels were measured in infected C57BL/6J and BALB/c mice. Intravenous injection of *P. falciparum*-derived hemozoin in malaria-free C57BL/6J mice induced inflammatory gene transcription in the liver, suggesting that hemozoin may be involved in the pathogenesis of malaria hepatopathy by inducing inflammation.

## Introduction

Malaria-associated hepatocellular dysfunction is commonly observed in combination with other organ involvement, both in adult and pediatric patients [1],[2]. It is a heterogeneous pathology with variable severity, as symptoms range from mild changes in liver function tests to severe liver failure, although the latter is uncommon. Hepatomegaly, jaundice, elevated liver enzymes e.g. alanine aminotransferase (ALT), and altered histopathological findings such as portal mononuclear cell infiltration are observed in varying degrees. Minimal fatty changes and liver cell necrosis are present, in line with low prevalence of steatosis and cholestasis. The most generalized feature observed throughout the liver is Kupffer cell hyperplasia with malaria pigment (hemozoin, Hz) deposition. Hz is released into the circulation together with the newly formed merozoites upon intraerythrocytic parasite replication and subsequent schizont rupture, and is rapidly removed by phagocytosis. The liver significantly contributes to phagocytosis of infected red blood cells (iRBC) and Hz, as evidenced by abundant pigment deposition on liver sections from patients [1],[2]. In mouse malaria models, the highest Hz levels are found in the liver compared to other organs including the spleen, and total Hz levels increase with disease severity [3]–[5]. In pediatric malaria infections, more Hz is found in the liver of patients deceased from cerebral pathology compared to parasitemic, comatose patients who died from non-malaria causes [1]. Furthermore, the incidence of hepatopathy is high in patients with severe malaria, e.g. with cerebral pathology or with malaria-associated acute respiratory distress syndrome (MA-ARDS) [6], and liver dysfunction seems to be correlated with acute renal failure [7]. The precise pathogenic mechanisms leading to jaundice and mild liver dysfunction are still poorly understood. In murine malaria models, liver damage is partly due to an imbalance in pro- and anti-inflammatory mediators in the liver, since restoring this balance diminishes pathology [8]–[13].

In the present study, we used three different parasite strains, i.e. *Plasmodium berghei* ANKA (*Pb*ANKA), *P. berghei* NK65 (*Pb*NK65) and *P. chabaudi* AS (*Pc*AS) and two different mouse strains (C57BL/6J and BALB/c) to obtain insights into the importance of parasite strain *versus* host genetics on liver pathology. In C57BL/6 mice *Pb*ANKA induces lethal neuropathology and also lung injury, whereas *Pb*NK65-infection results in lethal MA-ARDS [14]–[16]. BALB/c mice appear relatively resistant to both complications but succumb from hyperparasitemia with both parasite strains. In contrast, C57BL/6 mice successfully clear *Pc*AS-infections after a significant primary parasitemia peak and smaller recrudescences [17], whereas BALB/c mice are only partially resistant to *Pc*AS infection [18].

We have previously shown that *P. berghei* and *Pc*AS parasites produce different amounts of Hz, and that Hz is pathogenic in the lungs by inducing pulmonary inflammation [19]. In view of the high and differential levels of Hz in the liver of *P. berghei* or *Pc*AS-infected mice, we investigated whether infection with these parasites results in a similar type of hepatocellular dysfunction as observed in malaria patients. Furthermore, we determined the effect of Hz in such malaria-associated hepatopathy and compared this between mice infected with malaria parasites with a varying degree of virulence.

## Materials and Methods

### Ethics statement

All experiments were approved by the Animal Ethics Committee from the KU Leuven (License LA121251, Belgium).

### Mice and parasites

Male and female C57BL/6J and BALB/c mice (seven to eight weeks old; obtained from Janvier, Le Genest-Saint-Isle, France) were infected with 10^4^ iRBCs of the following parasite strains: *Pb*ANKA (clone Cl15cy1, a kind gift of Prof. C.J. Jansse, Leiden University Medical Center, The Netherlands), *Pb*NK65 or *Pc*AS (kind gifts of the late Prof. D. Walliker, University of Edinburgh, Scotland, UK) as described previously [19]. Peripheral parasitemia was determined by microscopic analysis after Giemsa staining (Sigma-Aldrich, Bornem, Belgium). Mice were sacrificed at the indicated time points after infection, blood was removed by cardiac puncture, incubated at room temperature for one hour and stored at 4°C until the next day when serum was collected and stored at −80°C until further analysis. Mice were perfused with phosphate-buffered saline (PBS, BioWhittaker, Lonza, Verviers, Belgium) and perfused livers were used to determine the Hz content by heme-enhanced chemoluminescence as described before [3]. The right lobe of the liver was embedded in Tissue-Tek O.C.T. Compound (Sakura Finetek Europe B.V., Alphen aan den Rijn, The Netherlands) and frozen for histological analysis. Alternatively, the right lobe was fixed in 4% paraformaldehyde and embedded in paraffin for histological analysis.

### Assessment of liver pathology

Liver function was determined by measuring serum ALT, aspartate aminotransferase (AST) (both obtained from Teco Diagnostics, California, USA) and glutamate dehydrogenase (GLDH, Diasys Diagnostic Systems, Germany) according to the manufacturers protocols. Liver histopathology was assessed by hematoxylin-eosin staining on paraffin-sections and immunohistochemistry. Immunohistochemistry was performed on cryosections with monoclonal anti-mouse F4/80 IgG2b (Cl:A3-1, Abcam, Cambridge, UK; dilution 1/50) or on paraffin-sections with anti-mouse Gr-1 (specific for Ly6G and Ly6C, clone RB6-8C5, Abcam; dilution 1/3000). Visualization was obtained by reaction with 3-amino-9-ethylcarbazole (AEC) chromogen (Dako, Heverlee, Belgium), which produces a red color in the presence of peroxide. Transmitted light images were taken through a 40×/1.4 oil Plan-Apochromat objective of an Axiovert 200 M microscope equipped with an AxioCamMRm camera (Zeiss, Göttingen, Germany). Image adjustments (Sigma and unsharp masking) were performed with the AxioVision 4.6 software. Alternatively, transmitted light images were taken through a 40×/0.65 or a 100×/1.25 oil N Plan objective of a Leica DM2000 microscope.

### Isolation of leukocytes from the liver

C57BL/6J mice were infected with *Pb*ANKA, *Pb*NK65 or *Pc*AS parasites or were left uninfected. At the indicated time points (day 10 for *Pb*NK65 and *Pc*AS and day 8 for *Pb*ANKA since these mice do not survive until day 10), mice were bled by heart puncture to collect serum and were systemically perfused with PBS. The caudate and quadrate lobes of the liver were stored at −80°C for Hz quantification, whereas the other parts of the liver were mechanically homogenized with the gentleMACS Dissociator (Miltenyi Biotec B.V., Leiden, The Netherlands) and passed through a 70 µm nylon cell strainer (BD Biosciences, Erembodegem, Belgium). The resulting cell suspensions were centrifuged and the pellets were resuspended in 40% Percoll solution (GE Healthcare, Uppsala, Sweden) and layered on a 72% Percoll solution. After centrifugation at 524×g for 20 minutes (break switched off), leukocytes at the interphase between both Percoll layers were collected. Subsequently, remaining RBCs were lysed and leukocytes were washed, enumerated with a Bürker hemacytometer, and analyzed by flow cytometry as described previously [19]. Leukocytes were stained with the following antibody combinations (from eBiosciences, Vienna, Austria, unless otherwise indicated): anti-mouse CD4 APC (clone GK1.5), anti-mouse CD8a PE (clone 53-6.7) and anti-mouse CD69 FITC (clone H1.2F3) for the detection of activated T cell subsets; anti-mouse CD11b APC (clone M1/70), anti-mouse F4/80 FITC (clone BM8), anti-mouse MHC-II PE (clone M5/114.15.2) and anti-mouse CD80 PE (clone 16-10A1) to determine the activation status of macrophages; or anti-mouse CD11b APC (clone M1/70), anti-mouse F4/80 FITC (clone BM8) and anti-mouse Ly-6G (Gr-1) PE (clone RB6-8C5) for neutrophil detection. After two additional wash steps, cells were analyzed with a FACScan flow cytometer with the CellQuest software (BD Biosciences). To exclude dying cells from the analysis, propidium iodide was added just before the samples were processed on the flow cytometer.

### Quantitative Reverse Transcription-Polymerase Chain Reaction (RT-PCR)

Quantitative RT-PCR was performed as described before [19]. In brief, after mechanical homogenization of perfused livers, total RNA was extracted and quantified. For each sample, cDNA was synthesized and quantitative PCR was performed on 25 ng and 12.5 ng cDNA with primer and probe sets from Applied Biosystems (Foster City, CA) or Integrated DNA Technologies (Leuven, Belgium). Data were normalized to 18S ribosomal RNA levels [20].

### Intravenous injection of *P. falciparum*-derived hemozoin

*P. falciparum*-derived Hz (*Pf*Hz) was prepared from cultured *P. falciparum*-iRBCs and treated with DNase as described before [19]. Contamination with endotoxin (lipopolysaccharide, LPS), was excluded with the E-Toxate assay (Limulus Amebocyte Lysate, gel solidification assay, Sigma), sensitivity threshold at 0.05–0.10 EU/mL. Different amounts of *Pf*Hz (100 to 900 nmol) in 200 µL of PBS or PBS alone were injected intravenously into C57BL/6J mice. After 6 hours, mice were sacrificed, livers were removed and the right lobe was used for *Pf*Hz quantification and quantitative RT-PCR analysis.

### Statistical analysis

*P*-values for the differences between 2 groups were calculated with the Mann-Whitney *U*-test, with the use of GraphPad Prism Software (GraphPad Software, San Diego, CA). The same software was used to calculate nonparametric Spearman correlation coefficients. A *p*-value less than 0.05 (*p*<0.05) was considered as statistically significant.

## Results

### Liver pathology is more severe in *Pc*AS-infected mice

**Figure 1:**
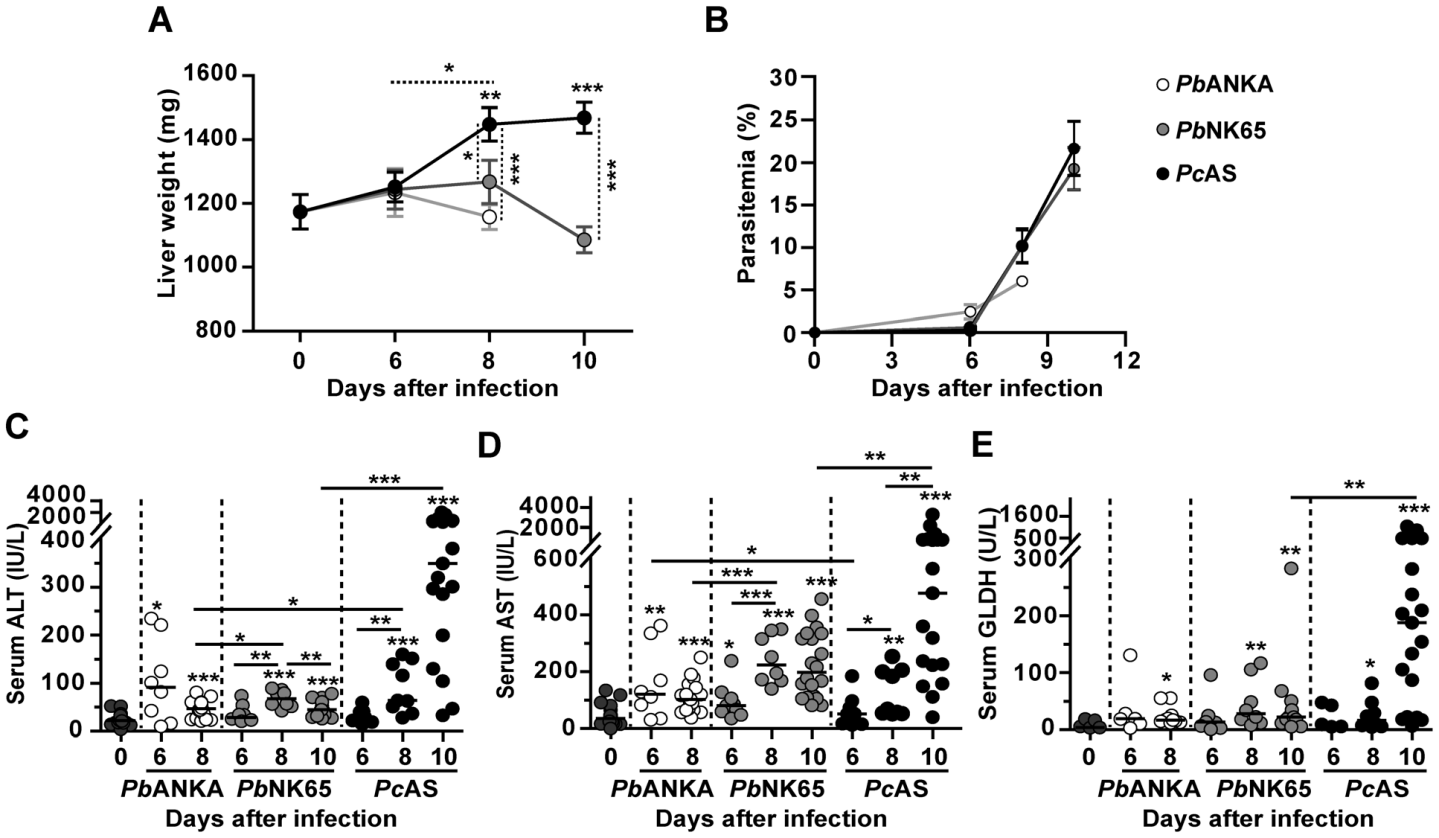
Liver pathology is more severe in *Pc*AS-infected mice than in *P. berghei*-infected mice. C57BL/6J mice were infected with *Pb*ANKA, *Pb*NK65 or *Pc*AS and sacrificed at different time points after infection. *Pb*ANKA-infected mice die around day 8, thus no data are available on day 10. (A) Weights of perfused livers. Means are indicated ± SEM (n = 8–21 for each condition). (B) Peripheral parasitemia levels. Means are indicated ± SEM (n = 8–21 for each group). (C–E) Serum levels of ALT (panel C), AST (panel D) and GLDH (panel E) levels. Data are pooled from two separate experiments with similar results. Each dot represents the results from an individual mouse. Small horizontal stripes between individual data points represent group medians and horizontal lines with asterisks on top indicate statistical differences between groups. Asterisks above individual data sets indicate statistical differences with the uninfected control group as follows: * *p*<0.05, ** *p*<0.01 and *** *p*<0.001.

To investigate whether hepatic Hz levels correlate with liver pathology, parameters of liver injury were investigated in C57BL/6J mice infected with three different *Plasmodium* strains with a varying degree of virulence i.e. *Pb*ANKA, *Pb*NK65 or *Pc*AS. Hepatomegaly was only observed in *Pc*AS-infected mice, whereas liver weights were slightly but not significantly decreased with *P. berghei* (Figure 1A). This suggested that more severe liver pathology developed in *Pc*AS-infected mice than in *P. berghei*-infected mice, despite similar peripheral parasitemia levels (Figure 1B).

Elevated serum liver enzymes like ALT and AST are an indication of possible liver injury. Serum ALT and AST levels increased 6 days after infection with *Pb*ANKA and *Pb*NK65 and decreased slightly thereafter (Figure 1C–D). In *Pc*AS-infected mice ALT and AST levels progressively increased from day 8 onwards and were significantly higher than in the serum of *Pb*NK65-infected mice 10 days after infection (*p*<0.0001 for ALT and *p* = 0.0073 for AST). The *Pb*ANKA mice succumbed 7–8 days after infection from cerebral pathology. GLDH is an enzyme found in the mitochondria of hepatocytes in the centrilobular areas of the liver and is an indicator of severe hepatocyte damage. Serum GLDH levels followed the same trend as the ALT and AST levels and started to increase significantly in the serum 8 days after infection with all three parasite strains and continued to increase at 10 days after infection in *Pc*AS-infected mice but not in *Pb*NK65-infected mice (*p* = 0.0072 for *Pc*AS *versus Pb*NK65 at day 10) (Figure 1E). Elevated serum levels of gamma glutamyl transpeptidase (γ-GT) and alkaline phosphatase (ALP) are indicative of bile duct damage and were also investigated, but these enzymes were undetectable (γ-GT) or decreased (ALP) during infection (data not shown).

Since host genetics are associated with differences in susceptibility to severe malaria, we also investigated liver pathology in BALB/c mice, which are relatively resistant to severe complications such as cerebral malaria and MA-ARDS ([16] and data not shown). As illustrated in Figure S1, serum ALT and AST levels (panels E, G) were similar or even lower in BALB/c mice upon *Pb*NK65 infection compared to C57BL/6J mice despite higher liver weights in BALB/c mice (panel C). Furthermore, BALB/c and C57BL/6J mice were equally susceptible to *Pc*AS-induced changes in the liver (panels F, H). When *Pc*AS parasitemia decreased after day 10 (as a consequence of antimalarial immunity) serum ALT and AST levels also decreased (Figure S1).

### Hepatic Hz levels correlate with elevated liver enzymes in *Pc*AS-infected mice

**Figure 2:**
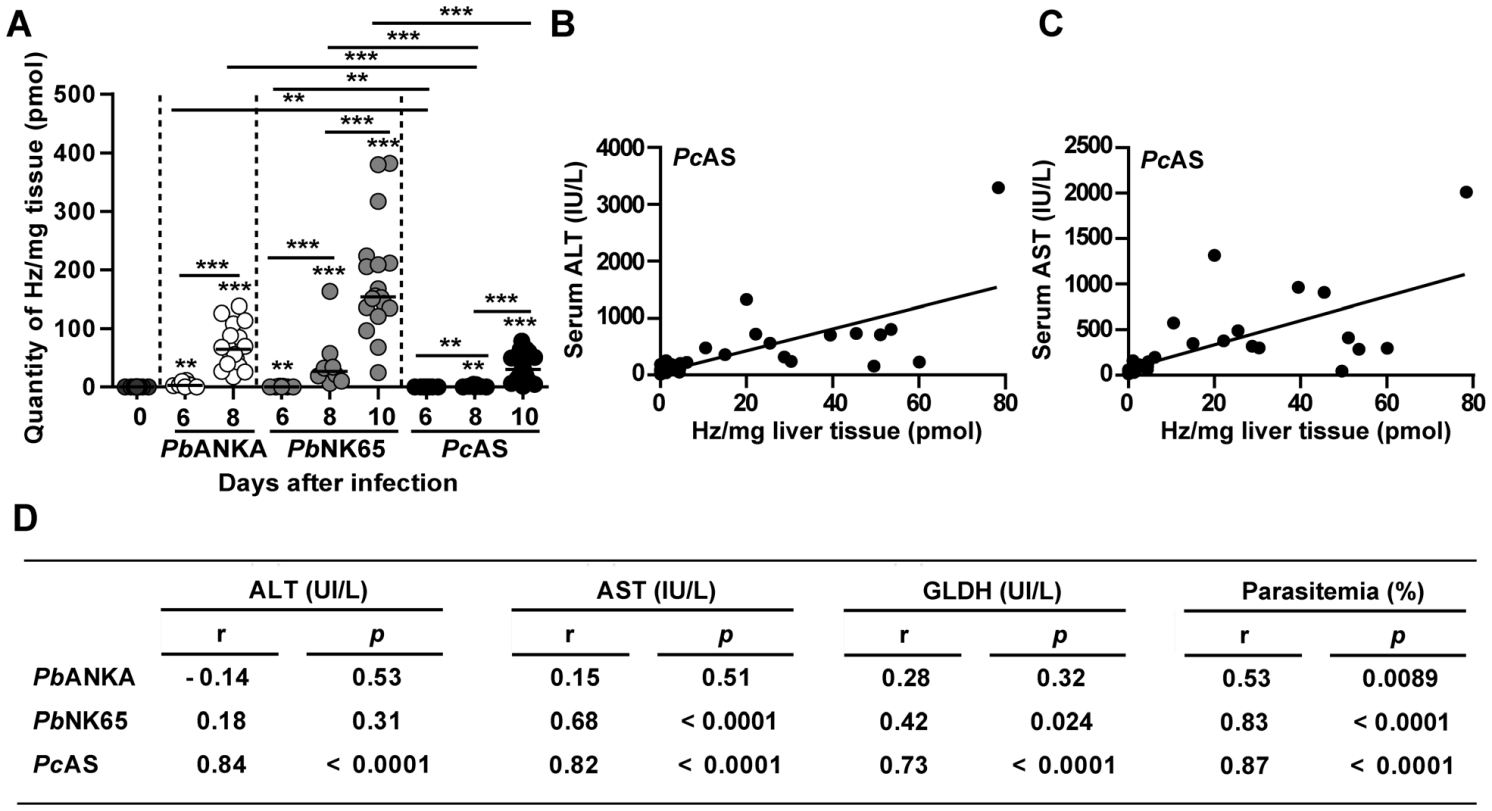
Hepatic hemozoin levels correlate with serum liver enzymes in *Pc*AS-infected mice. (A) Quantity of hemozoin (Hz)/mg liver tissue at different time points after infection with *Pb*ANKA, *Pb*NK65 or *Pc*AS. Data are from two separate experiments. Each dot represents the result from an individual C57BL/6J mouse. Horizontal bars between individual data points represent group medians and horizontal lines with asterisks on top indicate statistical differences between groups. ** *p*<0.01 and *** *p*<0.001. (B–C) Spearman correlations between hepatic Hz levels and serum ALT (panel B) or AST levels (panel C) in *Pc*AS-infected mice. (D) Spearman r and *p*-values between hepatic Hz levels and serum ALT, AST and GLDH levels with the different parasite strains.

Hz levels in the liver were determined in C57BL/6J mice infected with the abovementioned parasites. The amount of Hz/mg liver tissue increased progressively during infection (Figure 2A), suggesting that when infection progressed, more Hz was released into the circulation and trapped in the liver. Furthermore, a positive correlation was found between Hz levels and peripheral parasitemia levels with all three parasite strains (Figure 2D). At similar time points after infection and at comparable peripheral parasitemia levels (see Figure 1B) no major differences were found in the amount of Hz in livers from *Pb*ANKA and *Pb*NK65-infected mice, whereas livers from *Pc*AS-infected mice contained significantly less Hz. Also in BALB/c mice Hz levels progressively increased, correlated with peripheral parasitemia (r = 0.78 and *p*<0.0001 for *Pb*NK65; r = 0.94 and *p*<0.0001 for *Pc*AS), and were significantly higher with *Pb*NK65 compared to *Pc*AS (*p*<0.05 for all time points) (Figure S1).

The amount of Hz/mg liver tissue strongly correlated with the level of liver enzymes measured in the serum of *Pc*AS-infected C57BL/6J mice, as shown in Figure 2 panels B–D. No correlations were found in *Pb*ANKA-infected mice (Figure 2D). In *Pb*NK65-infected mice, a positive correlation was found between hepatic Hz and AST levels, whereas the correlation between GLDH levels and Hz had a low Spearman r coefficient. Together these data suggest that Hz is associated with liver injury in *Pc*AS infection but not in *P. berghei*-infected mice, although the amounts of Hz in the liver are considerably higher with *P. berghei* compared to *Pc*AS. Since the liver pathology depends on the parasite strain and is only marginally affected by the mouse strain, only C57BL/6J mice were used for the subsequent experiments in this manuscript.

### Marked inflammation in livers of *Pc*AS-infected mice

**Figure 3:**
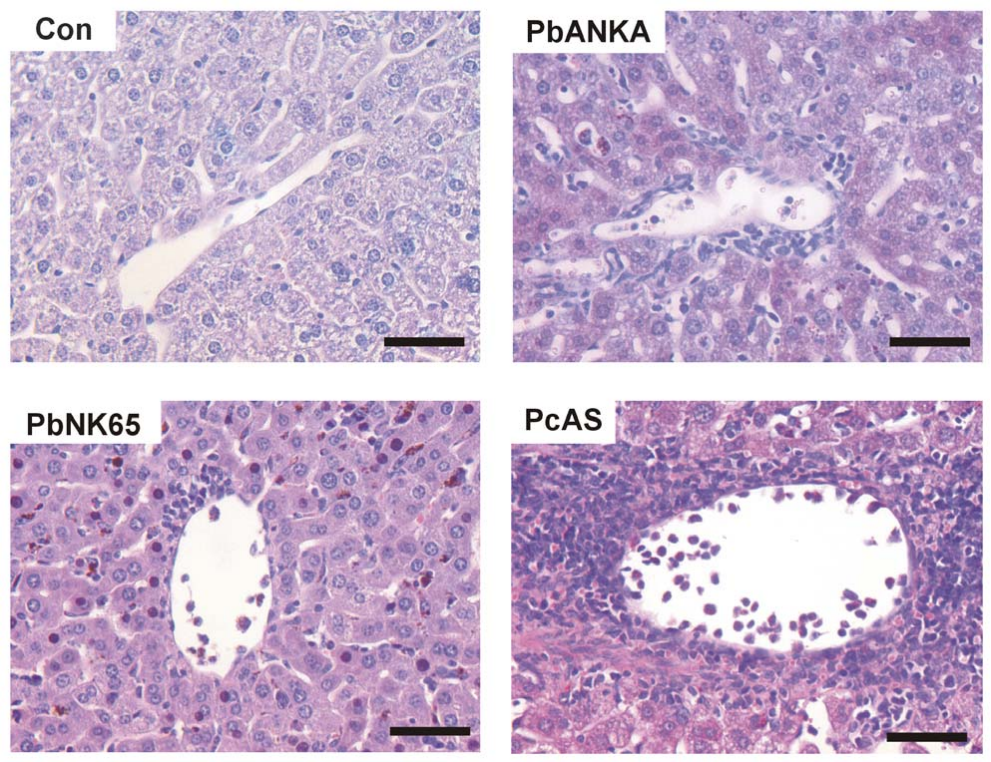
Massive inflammatory infiltrates in livers of *PcAS*-infected mice. Paraffin-embedded sections were prepared from livers of uninfected C57BL/6J mice (Con) and from mice infected with *Pb*ANKA (day 8), *Pb*NK65 (day 10) or *Pc*AS (day 10), and stained with hematoxylin–eosin. Representative images are shown (scale bars, 50 µm).

To analyze the underlying mechanism of the increased liver enzyme levels detected in peripheral blood, liver morphology was investigated on hematoxylin-eosin-stained paraffin sections from perfused livers obtained from mice infected with the different parasite strains. No major morphological changes were noticeable in the liver architecture, since only minor single cell necrosis was detected, whereas fibrosis, steatosis, hepatocyte enlargement, bile duct damage, obstruction of blood vessels, sinuses or bile ducts, were not observed with this method. We did observe massive perivenular, parenchymal and, to a lesser extent, periportal mononuclear cell infiltration in livers from *Pc*AS-infected C57BL/6J mice at day 10 after infection (Figure 3). Such inflammatory infiltrates were strikingly less abundant in livers from *Pb*NK65 (day 10) or *Pb*ANKA-infected mice (day 8) and were presumably the main cause of the hepatomegaly with *Pc*AS.

### Hz in the liver is clustered in macrophages

**Figure 4:**
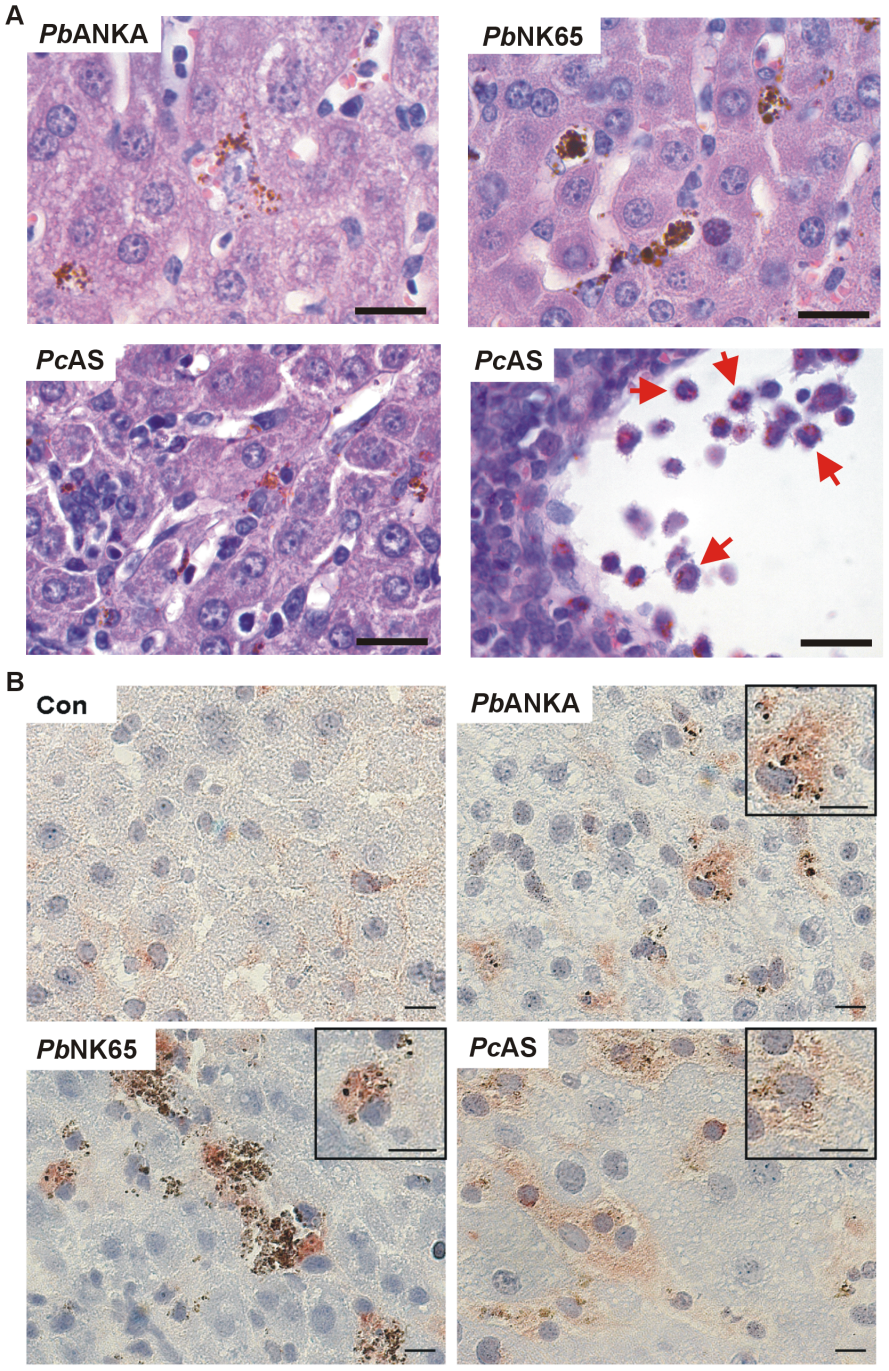
Hepatic hemozoin is localized in macrophages. (A) Higher magnification of clusters of hemozoin (Hz, brown crystals) in hematoxylin-eosin stained paraffin sections of livers from C57BL/6J mice mice infected with *Pb*ANKA (day 8), *Pb*NK65 (day 10) or *Pc*AS (day 10). The red arrows indicate rare Hz-containing neutrophils. Additional images are shown in Figure S2. Representative images are shown (scale bars, 20 µm). (B) Cryosections were stained with anti-F4/80 monoclonal antibodies (red color) to identify monocytes/macrophages. Insets show clusters of Hz inside F4/80^+^ cells. Representative images are shown (scale bars, 10 µm).

Since high quantities of Hz were detected in livers from infected mice and we previously showed that Hz in lungs is derived from both infected erythrocytes present in the microvasculature and from phagocytes that had ingested Hz [19], we investigated the cells containing the Hz in the liver in more detail by histology. Livers from *Pb*NK65-infected mice 10 days after infection contained large clusters of Hz, which were larger and more abundant than those observed in *Pb*ANKA (day 8) or *Pc*AS-infected mice (Figure 4A), corroborating the Hz quantification data (see Figure 2). These Hz clusters were mainly observed in sinusoidal macrophages, whereas only limited numbers of Hz-containing neutrophils were observed, which were exclusively found in the lumen of blood vessels (Figure 4A). Some Hz was also visible in marginating phagocytes, whereas endothelial cells did not contain any detectable Hz (Figure S2). Remarkably, hardly any iRBCs containing Hz were detected in these sections, indicating that hepatic Hz at the moment that these mice were sacrificed is almost exclusively located in the phagocytic compartment.

To confirm that the Hz-containing cells are indeed macrophages, immunohistochemistry was performed with an anti-F4/80 antibody. Hz crystals (brown) colocalized with F4/80 staining (red), indicating that Hz is mostly situated inside macrophages (Figure 4B). Interestingly, hepatic F4/80-staining was more widely distributed in *Pc*AS-infected mice, suggesting that monocyte/macrophage numbers are higher in *Pc*AS livers than in *P. berghei* livers. Gr-1 immunohistochemical staining of a section from a *Pc*AS-infected liver indicated that the Hz-containing macrophages were also Gr-1^+^. Gr-1^hi^ monocytes were also detected in the liver, these contained only very limited or no Hz (Figure S3).

### More extensive mononuclear cell infiltration in *Pc*AS-infected livers

**Figure 5:**
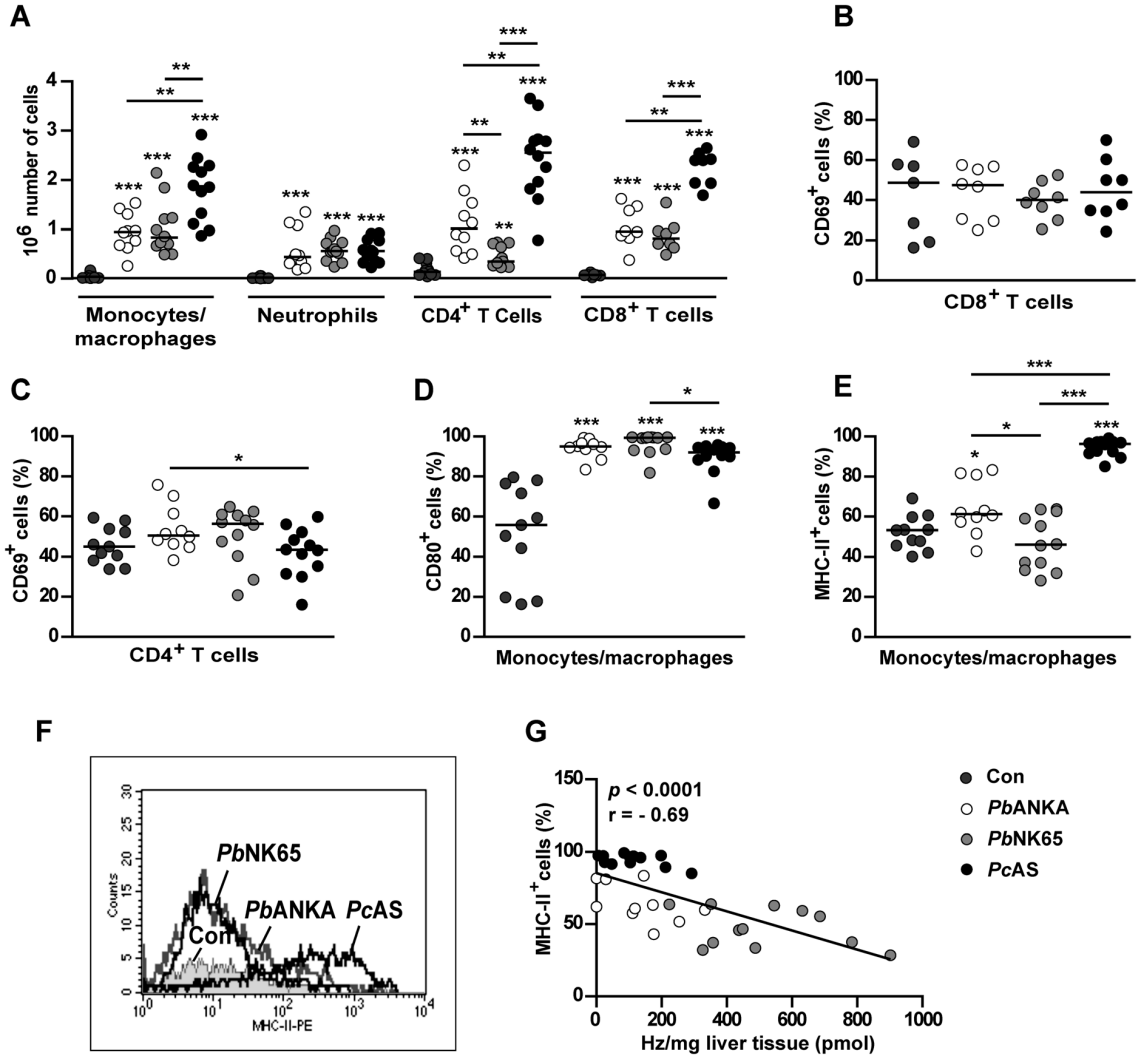
Inflammatory cell recruitment to the liver. C57BL/6J mice were left uninfected (Con) or were infected with *Pb*ANKA, *Pb*NK65 or *Pc*AS and hepatic leukocytes were analyzed 8 (*Pb*ANKA) or 10 (*Pb*NK65, *Pc*AS) days after infection by flow cytometry. (A) Total numbers of monocytes/macrophages (F4/80^+^CD11b^+^), neutrophils (F4/80^−^CD11b^+^Gr-1^+^), CD4^+^ T cells and CD8^+^ T cells in the liver. Proportion of CD8^+^ T cells (B) or CD4^+^ T cells (C) expressing the early activation marker CD69. Proportion of monocytes/macrophages expressing CD80 (D) or MHC class II (E). Data are pooled from three separate experiments with similar results. (F) Histogram showing the MHC-II expression on F4/80^+^CD11b^+^ cells from Con, *Pb*ANKA, *Pb*NK65 or *Pc*AS-infected mice. (G) Spearman correlations between hepatic hemozoin (Hz) amounts and the proportion of monocytes/macrophages expressing MHC class II in infected mice. Spearman r and *p*-values are indicated.

To investigate hepatic cell infiltrations in more detail, leukocytes were isolated from the liver and analyzed by flow cytometry. With the three different parasite strains, higher numbers of monocytes/macrophages (F4/80^+^CD11b^+^), CD4^+^ T cells and CD8^+^ T cells were detected in infected *versus* uninfected control mice (Fig. 5A). Livers from *Pc*AS-infected mice contained significantly more monocytes/macrophages, CD4^+^ T cells and CD8^+^ T cells compared to *Pb*ANKA and *Pb*NK65-infected livers, whereas neutrophil (F4/80^−^CD11b^+^Gr-1^+^) numbers were similarly increased with the three parasite strains. These data thereby confirm our histological data stating that the inflammatory infiltrates in livers from *Pc*AS-infected mice are mainly due to mononuclear cells.

To analyze the activation phenotype of the different cell subsets, additional surface markers were determined. The percentage of T cells expressing CD69 was merely similar in the liver of uninfected and infected mice (Figure 5B–C). Furthermore, almost all hepatic monocytes/macrophages from infected mice expressed higher CD80 levels than those from uninfected mice, without significant differences among the three parasite strains, indicating that monocytes/macrophages are similarly activated by infection with the three parasites (Figure 5D). However, whereas almost all monocytes/macrophages derived from *Pc*AS livers expressed major histocompatibility complex (MHC) class II molecules, the percentages of cells expressing this marker was not very different between *P. berghei* infected and uninfected livers (Figure 5E–F). Moreover, a strong negative correlation was found between hepatic Hz levels and both the number of MHC-II^+^ F4/80^+^CD11b^+^ cells (*p* = 0.0022 and r = −0.51) and the percentage of F4/80^+^CD11b^+^ cells expressing MHC class II (*p*<0.0001 and r = −0.69) (Figure 5G).

### Hz, serum liver enzymes and hepatic inflammation are tightly correlated in *Pc*AS-infected mice

**Figure 6:**
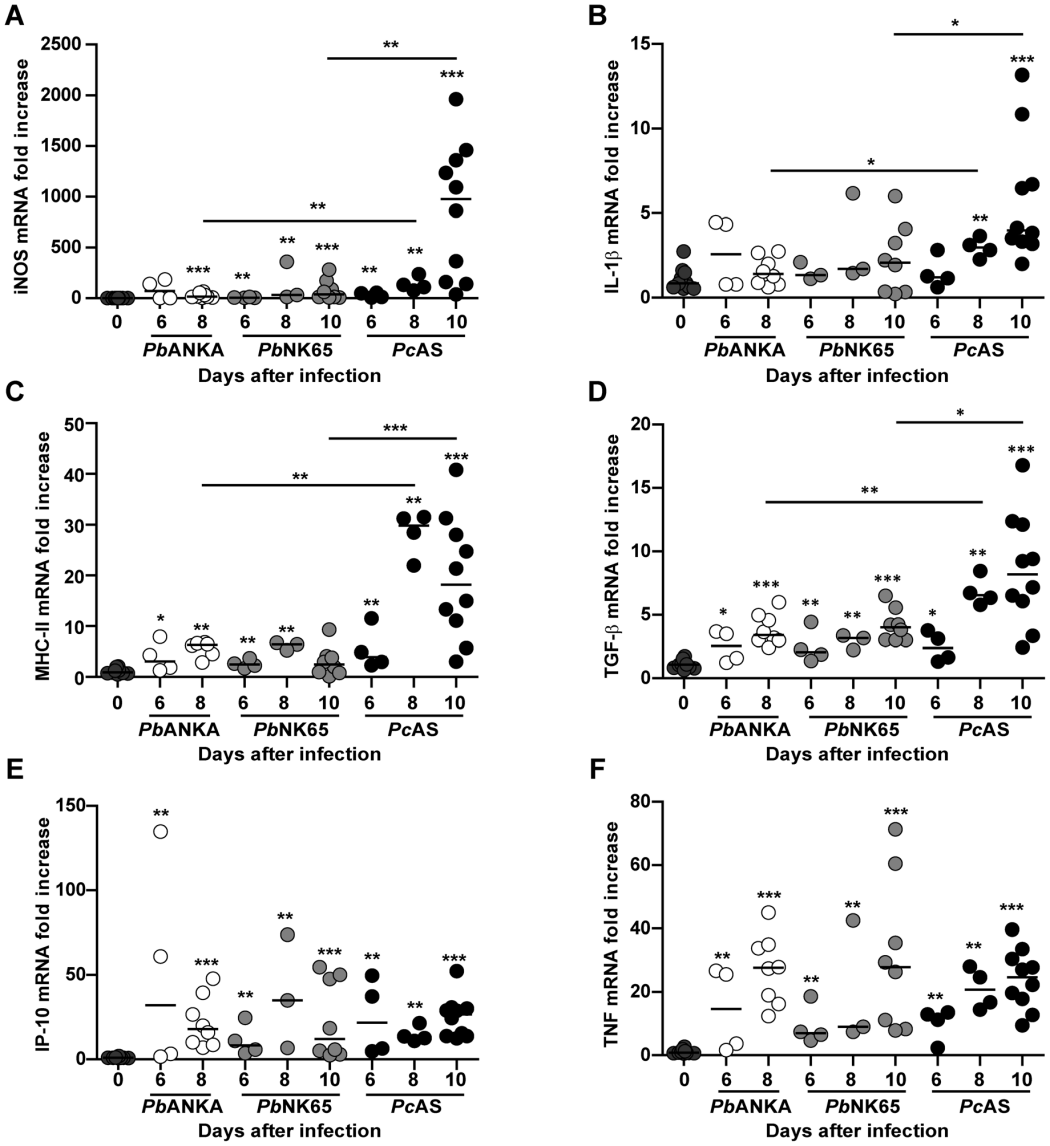
Transcription of inflammatory mediators in the liver of infected mice. C57BL/6J mice were left uninfected or were infected with *Pb*ANKA, *Pb*NK65 or *Pc*AS and hepatic mRNA expression levels of 20 different cytokines and other inflammatory mediators were determined at different time points after infection (6, 8 and 10 days) by quantitative RT-PCR and normalized to 18S ribosomal RNA levels. In this figure, data are shown for iNOS (A), IL-1β (B), MHC class II (C), TGF-β (D), IP-10 (E) and TNF (F), the data of the other mediators are shown in table 1. Data are from two separate experiments. Each dot represents the result from an individual mouse. Horizontal bars between individual data points represent group medians and horizontal lines with asterisks on top indicate statistical differences between groups. Asterisks above individual data sets indicate statistical differences with the uninfected control group: * *p*<0.05, ** *p*<0.01 and *** *p*<0.001.

The expression levels of twenty different cytokines, chemokines, and other inflammatory mediators was measured in the liver by quantitative RT-PCR (Figure 6 and Table 1). Most mediators, e.g. tumor necrosis factor (TNF), interferon gamma-induced protein-10 (IP-10/CXCL10), and monocyte chemoattractant protein-1 (MCP-1/JE/CCL2) were similarly induced upon infection with the three parasite strains, whereas the mRNAs of lymphotoxin-α (LT-α) and interleukin (IL)-1β were only significantly increased in *Pc*AS livers. However, major differences were found between *Pc*AS and both *P. berghei* strains for e.g. transforming growth factor-β (TGF-β), inducible nitric oxide synthase (iNOS) and MHC class II, of which the mRNA levels were highest in *Pc*AS livers.

**Table 1:** mRNA expression levels of inflammatory mediators in the liver from C57BL/6J mice infected with *Pb*ANKA, *Pb*NK65 or *Pc*AS.

	Con	*Pb*ANKA d6	*Pb*ANKA d7–8	*Pb*NK65 d6	*Pb*NK65 d8	*Pb*NK65 d9–10	*Pc*AS d6	*Pc*AS d8	*Pc*AS d10
**TNF**	0.83 (0.62–2.74)	25.57** (3.65–26.71)	27.68*** (12.42–44.99)	6.98** (6.49–18.64)	9.05** (7.46–42.55)	27.90*** (7.88–71.34)	12.10** (2.37–13.59)	20.73** (14.46–28.08)	24.65*** (9.49–39.73)
**IP-10**	0.92 (0.72–1.92)	60.96** (3.24–134.82)	17.88*** (8.55–39.35)	8.33** (3.64–24.62)	34.98** (6.79–73.71)	12.08*** (2.35–50.00)	21.81** (4.73–49.58)	13.07** (10.90–21.46)	26.53*** (12.47–52.31)
**MCP-1**	0.80 (0.56–1.92)	53.59 (1.29–62.20)	12.28*** (3.82–33.77)	3.91** (2.03–13.03)	5.59** (4.12–62.65)	13.13*** (3.32–79.16)	5.09* (1.17–6.05)	4.89** (2.62–5.13)	19.96*** (5.42–35.11)
**KC**	0.71 (0.42–3.3)	26.08 (1.06–37.15)	5.34** (1.24–58.06)	1.64 (0.54–6.31)	6.58* (3.16–43.55)	24.02*** (1.50–99.38)	2.05 (1.06–2.48)	1.80 (1.40–2.38)	6.46*** (3.23–7.99)
**IL-10**	0.99 (0.55–2.29)	25.50** (3.55–33.05)	60.02*** (40.51–101.01)	5.14** (4.24–9.25)	121.80** (3.60–133.16)	49.03*** (8.38–249.14)	4.70** (1.64–84.23)	19.04** ‡‡ (9.27–25.55)	31.47*** (3.08–57.09)
**iNOS**	0.90 (0.53–1.95)	140.53 (1.81–184.80)	16.23*** (5.37–63.95)	5.47** (3.63–11.15)	32.55** (13.17–360.27)	37.79*** (6.87–281.55)	30.56** (6.27–53.29)	120.61** ‡‡ (77.73–237.33)	976.86*** †† (37.50–1961.29)
**NOX2**	1.01 (0.57–2.25)	6.05* (1.65–6.53)	8.11*** (5.57–9.30)	4.27** (3.56–8.23)	6.82 (3.94–12.13)	8.05*** (3.84–10.67)	3.82** (1.61–7.89)	10.04** ‡‡ (8.55–14.08)	11.02*** (2.66–13.54)
**Perforin**	0.85 (0.48–2.58)	28.09 (1.61–28.29)	28.68*** (26.63–33.90)	7.57** (4.94–12.91)	52.36** (6.41–74.35)	32.45*** (14.63–103.65)	6.98** (1.85–41.35)	29.27** (23.53–36.49)	32.06*** (8.71–69.73)
**Hmox1**	0.99 (0.67–1.60)	14.00** (1.99–24.07)	21.00*** (15.61–29.77)	3.13** (2.40–5.94)	16.23** (3.37–25.20)	13.17*** (5.21–37.00)	3.79* (0.97–27.15)	15.99** (11.95–25.81)	30.07*** (5.19–72.20)
**IL-12**	0.98 (0.55–1.90)	3.24** (2.55–4.23)	13.14*** (4.46–19.86)	4.17** (2.02–4.45)	11.03* (1.82–11.76)	7.83*** (3.54–16.58)	4.24** (2.65–18.62)	10.57** (7.28–15.37)	6.57*** (2.14–20.33)
**ICAM-1**	1.02 (0.54–2.06)	9.39** (1.58–12.71)	7.12*** (5.73–10.04)	3.39** (2.21–8.52)	6.85** (6.34–16.06)	8.34*** (3.07–15.95)	4.76** (2.08–7.96)	8.14** (6.41–8.79)	9.48*** (5.83–14.96)
**FasL**	1.02 (0.65–2.14)	5.52 (1.15–5.87)	9.10*** (6.63–12.85)	3.19** (2.75–5.48)	9.50** (3.12–12.56)	8.46*** (3.28–12.35)	2.78* (1.21–8.27)	10.32** (9.28–11.73)	11.89*** (3.85–22.42)
**IFN-γ**	0.88 (0.61–2.00)	17.03* (2.14–34.77)	50.59*** (24.92–104.63)	4.93** (3.87–17.77)	27.20* (22.25–71.28)	34.92*** (10.33–128.79)	33.08** (7.85–43.27)	95.49** ‡ (86.26–246.59)	91.01*** (50.63–129.43)
**MMP-9**	0.83 (0.65–2.19)	5.83** (1.05–7.33)	1.70 (1.30–2.51)	1.65 (1.43–2.52)	7.86*** (1.00–16.58)	3.50 (2.21–9.58)	1.99 (0.79–3.40)	6.48** ‡‡ (4.05–10.43)	4.31*** (1.67–49.41)
**LT-α**	1.08 (0.49–1.15)	11.21 (0.98–13.64)	3.61 (0.76–13.51)	4.64 (3.80–4.67)	9.65 (4.28–14.39)	3.72 (0.74–5.67)	4.88 (0.39–5.94)	8.65* (6.37–11.80)	3.55** (1.28–8.23)
**IL-6**	0.98 (0.58–2.13)	19.24 (1.00–24.13)	2.54** (1.17–3.78)	1.72 (1.28–6.14)	1.42* (1.41–17.05)	3.56 (0.58–10.59)	1.51 (0.41–2.40)	1.09‡ (0.99–1.73)	7.50*** (0.66–25.69)
**IL-1β**	0.86 (0.54–2.75)	4.35 (0.78–4.46)	1.41 (0.61–2.74)	1.34 (1.12–1.34)	1.71 (1.46 6.18)	2.08 (0.21–6.01)	1.21 (0.63–2.83)	2.97** ‡ (2.27–3.65)	3.99*** † (2.01–13.18)
**IL-4**	1.02 (0.40–2.41)	6.64 (0.93–7.62)	1.73* (1.04–2.47)	1.65 (0.60–2.03)	1.19 (1.02–3.57)	0.42 (0.22–4.77)	1.06 (0.65–1.42)	4.06** ‡‡ (3.86–4.23)	5.78*** ††† (1.20–10.33)
**TGF-β**	1.08 (0.64–1.74)	3.49* (1.24–3.68)	3.42*** (2.42–5.99)	2.07** (1.37–4.43)	3.16** (2.24–3.37)	4.00*** (3.02–6.50)	2.39* (1.32–3.77)	6.54** ‡‡ (5.79–8.47)	8.19*** † (2.43–16.78)
**MHC-II**	0.89 (0.51–2.10)	4.31* (1.85–4.31)	6.33** (2.85–6.82)	2.45** (1.70–3.67)	6.40** (5.24–6.76)	2.46 (0.14–9.33)	3.92** (2.28–11.54)	29.86** ‡‡ (21.96–31.51)	18.17*** ††† (3.01–31.28)

Median values are shown for each group (n = 3–10) and min-max values are indicated between parentheses. Statistically different from control (Con) mice:

* *p*<0.05;

** *p*<0.01;

*** *p*<0.001;

statistically different from *Pb*NK65 at similar time points:

† *p*<0.05;

†† *p*<0.01;

††† *p*<0.001;

statistically different from *Pb*ANKA at similar time points:

‡ *p*<0.05;

‡‡ *p*<0.01;

FasL, Fas ligand; Hmox1, heme oxygenase 1; ICAM-1, intercellular adhesion molecule-1; IFN-γ, interferon-gamma; IL-, interleukin-; iNOS, inducible nitric oxide synthase; IP-10, interferon gamma inducible protein-10; KC, keratinocyte-derived chemokine; LT-α, lymphotoxin-alpha; MCP-1, monocyte chemotactic protein-1; MHC-II, major histocompatibility complex class II; MMP-9, matrix metalloproteinase-9/gelatinase B; NOX2, NADPH oxidase 2; TGF-β, transforming growth factor-beta; TNF, tumor necrosis factor.

The association between Hz, hepatocyte damage and the induction of these mediators was determined by Spearman correlation analysis (Table 2). In *Pc*AS-infected mice we found a positive correlation with both the amount of Hz/mg liver tissue and ALT and AST levels and the mRNA expression levels of fourteen mediators, including IL-1β, IL-10, TNF, TGF-β, MCP-1, keratinocyte chemotactic protein (KC/CXCL1) and iNOS, whereas little or no correlation existed with mRNA levels of IL-4, IL-12, LT-α, IFN-γ, IP-10, and MHC class II. Strikingly, little or no mutual correlations were observed between the mRNA expression of inflammatory mediators and both Hz and liver enzymes in *Pb*ANKA or *Pb*NK65-infected mice.

**Table 2:** Spearman correlations between Hz, liver enzymes and mRNA expression levels of inflammation-associated proteins.

	Hz/mg liver tissue				ALT				AST			
	*Pb*ANKA	*Pb*NK65	*Pc*AS	All	*Pb*ANKA	*Pb*NK65	*Pc*AS	All	*Pb*ANKA	*Pb*NK65	*Pc*AS	All
**TNF**	0.76	0,28	0,64**	0,44**	0,37	0,18	0,83****	0,45**	0,50	0,21	0,83****	0,44**
**IP-10**	0,25	−0,24	0.08	0,11	0,41	0,27	0,11	0,32*	0,25	0,04	0,03	0,13
**MCP-1**	0,25	0,29	0,67**	0,47**	0,39	0,32	0,69**	0,49***	0,30	0,30	0,67**	0,50***
**KC**	0.38	0,42	0.78***	0,64****	0,22	0,39	0,76***	0,78*	0,22	0,42	0,76***	0,60****
**IL-10**	0,59	0,36	0,72***	0,60****	0,45	0,11	0,58*	0,31*	0,45	0,17	0,64**	0,45**
**iNOS**	0,19	0.38	0,79***	0,19	0,45	0,51*	0,90****	0,69****	0,28	0,50	0,90****	0,50***
**NOX2**	0,08	0,15	0,52*	0,27	0,50	0,08	0,69**	0,53***	0,30	0,08	0,65**	0,47**
**Perforin**	0,15	0,45	0,55*	0,43**	0,72*	0,21	0,57*	0,45**	0,55	0,29	0,63**	0,53***
**Hmox1**	−0,06	0,38	0,79****	0,41**	0,54	0,11	0,79***	0,55***	0,32	0,14	0,83****	0,53***
**IL-12**	0,4	0.41	0,44	0,37*	0,32	−0,13	0,40	0,22	0,31	0,14	0,44	0,31*
**ICAM-1**	0,34	0,007	0.69**	0,3*	0,43	0,25	0,84****	0,53***	0,30	0,18	0,82****	0,47**
**FasL**	0,38	0,17	0,66***	0,36*	0,28	0,11	0,78***	0,58****	0,47	0,09	0,79***	0,54***
**IFN-γ**	0,82 **	0,39	0,32	0,17	0,43	0,3	0,5 *	0,53 ***	0,5	0,33	0,54 *	0,37 *
**MMP-9**	0	0,3	0,5*	0,22	0,59	0,17	0,60*	0,42**	0,62*	0,28	0,59*	0,46**
**LT-α**	−0,54	−0,13	−0,04	−0,13	−0,04	−0,17	0,18	0,19	−0,25	−0,13	0,05	0,05
**IL-6**	0,24	−0,27	0,81****	0,26	0,39	−0,08	0,79***	0,42**	0,36	−0,22	0,78***	0,42**
**IL-1β**	0,17	−0,27	0,75***	0,02	0,3	−0,04	0,91****	0,55***	0,28	−0,22	0,92****	0,38*
**IL-4**	−0,49	−0,45	0,44	−0,24	0,04	0,03	0,46	0,43**	−0,02	−0,20	0,43	0,06
**TGF-β**	0,33	0.37	0,74***	0,24	0,71*	0,29	0,80***	0,72****	0,89***	0,18	0,81****	0,54***
**MHC-II**	−0,03	0.13	0,29	−0,17	0,81*	0,34	0,55*	0,64****	0,45	0,32	0,53*	0,22

* *p*<0.05;

** *p*<0.01;

*** *p*<0.001;

**** *p*<0.0001;

FasL, Fas ligand; Hmox1, heme oxygenase 1; ICAM-1, intercellular adhesion molecule-1; IFN-γ, interferon-gamma; IL-, interleukin-; iNOS, inducible nitric oxide synthase; IP-10, interferon gamma inducible protein-10; KC, keratinocyte-derived chemokine; LT-α, lymphotoxin-alpha; MCP-1, monocyte chemotactic protein-1; MHC-II, major histocompatibility complex class II; MMP-9, matrix metalloproteinase-9/gelatinase B; NOX2, NADPH oxidase 2; TGF-β, transforming growth factor-beta; TNF, tumor necrosis factor.

### Injection of malaria-free C57BL/6J mice with *P. falciparum*-derived Hz mimics the inflammatory profile observed in the liver

**Figure 7:**
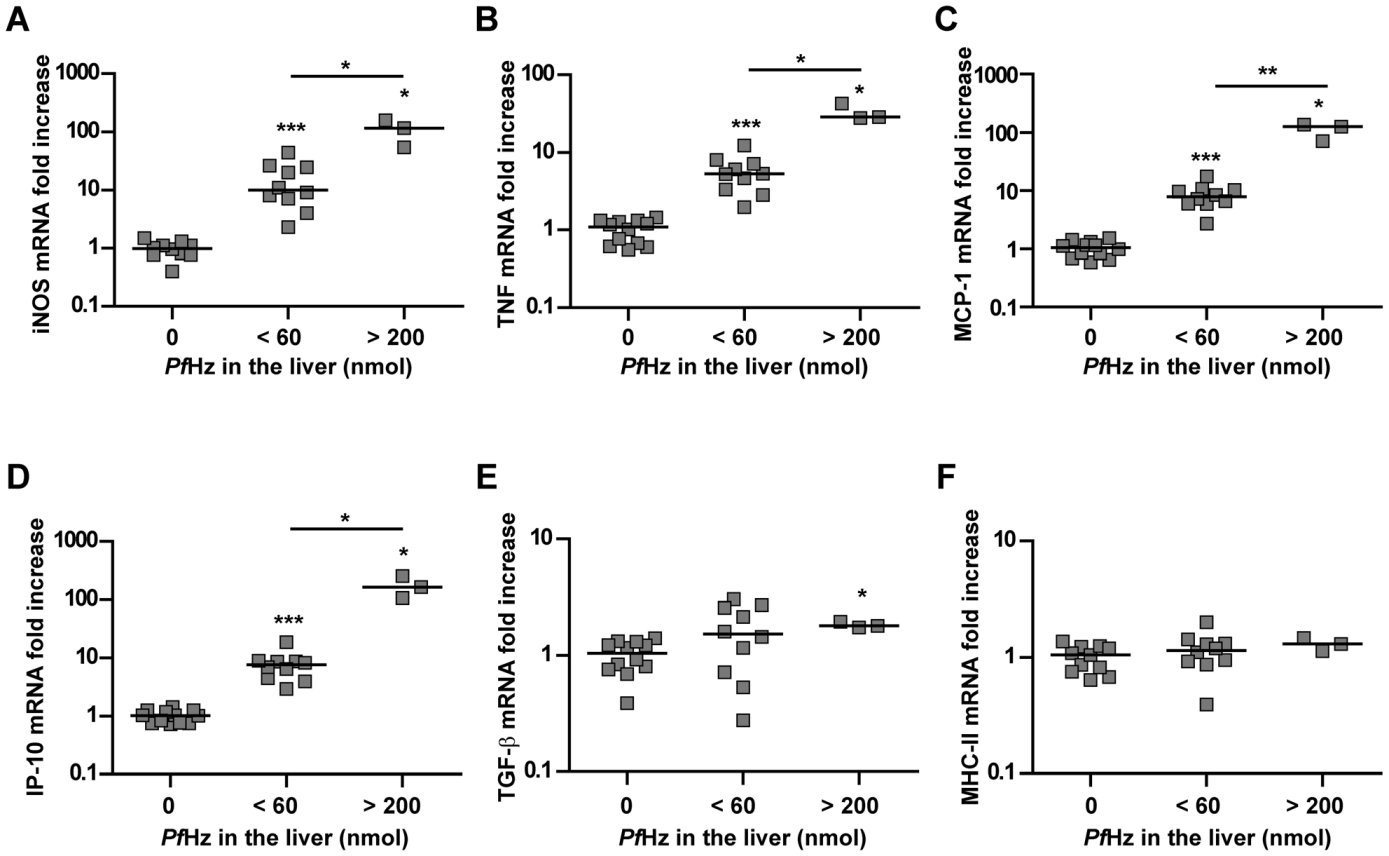
Injection of *Pf*Hz induces the expression of inflammatory mediators in the liver. Naive malaria-free C57BL/6J mice were injected intravenously with different amounts of *Pf*Hz and dissected 6 hours later. Hepatic mRNA expression levels are shown for iNOS (A), TNF (B), MCP-1 (C), IP-10 (D), TGF-β (E) or MHC class II (F) as determined by quantitative RT-PCR and normalized to 18S ribosomal RNA levels. Data for other mediators are shown in table 3. Data were stratified according to the total amount of *Pf*Hz measured in the liver. Horizontal bars between individual data points indicate group medians. Horizontal lines with asterisks on top indicate statistical differences between groups. Asterisks above individual data sets indicate statistical differences with the vehicle-injected control group. * *p*<0.05, ** *p*<0.01 and *** *p*<0.001.

To confirm the causal relation between Hz and inflammation in the liver, *Pf*Hz was injected intravenously in malaria-free C57BL/6J mice. Mice were sacrificed 6 hours after injection and hepatic *Pf*Hz levels and the mRNA expression of inflammatory mediators was determined. When stratifying the data according to hepatic *Pf*Hz levels, we observed that *Pf*Hz dose-dependently induced the expression of nine of these mediators, i.e. IL-6, TNF, interferon-γ (IFN-γ), MCP-1, IP-10, KC, iNOS, intercellular adhesion molecule-1 (ICAM-1) and perforin in the liver (Figure 7 and Table 3). IL-1β, heme oxygenase 1 (Hmox1), TGF-β, Fas ligand (FasL), matrix metalloproteinase-9 (MMP-9)/gelatinase B and NADPH oxidase 2 (NOX2) were also induced but the mRNA levels did not change significantly with varying amounts of hepatic *Pf*Hz. IL-10, LT-α and MHC class II mRNA levels were not significantly induced at this time point. These results demonstrate that *Pf*Hz injection induces the expression of several cytokines, chemokines and other inflammatory mediators in the liver that were associated with hepatic Hz levels and serum ALT and AST levels in *Pc*AS-infected mice.

**Table 3:** Hepatic mRNA expression of inflammatory mediators 6 h after intravenous injection of *Pf*Hz.

	PBS	*Pf*Hz Li <70 nmol	*Pf*Hz Li >200 nmol
**TNF**	1.09 (0,56–1,46)	5.32*** (1,98–12,37)	28.76* †† (28,05–43,18)
**IP-10**	1.01 (0,72–1,42)	7.59*** (2,91–18,77)	163.10* † (107,49–253,59)
**MCP-1**	1.04 (0,59–1,52)	7.77*** (2,72–17,58)	125.98* †† (70,95–136,76)
**KC**	1.01 (0,39–2,09)	1.85** (0,42–3,85)	5.46* †† (4,06–6,30)
**IL-10**	1.07 (0,32–1,47)	1.35 (0,56–3,07)	0.73 (0,51–0,83)
**iNOS**	0.98 (0,40–1,48)	10.04*** (2,31–24,58)	116.68** †† (54,09–160,30)
**NOX2**	1.01 (0,47–1,49)	1.42 (0,17–2,99)	1.95* (1,94–2,07)
**Perforin**	0.96 (0,54–1,37)	1.75** (0,81–3,30)	6.02* †† (5,46–6,02)
**Hmox1**	1.02 (0,52–1,30)	1.74* (0,31–2,98)	2.25* (1,64–2,70)
**ICAM-1**	0.96 (0,76–1,44)	2.38*** (0,92–3,30)	16.20* †† (7,89–17,77)
**FasL**	1.03 (0,30–1,70)	1.77 (0,25–2,35)	2.24* (2,12–2,83)
**IFN-γ**	0.93 (0,22–2,24)	2.16** (1,08–6,72)	60.37* †† (55,15–64,30)
**MMP-9**	0.95 (0,79–1,31)	1.18 (0,20–1,74)	1.55* (1,18–1,68)
**LT-α**	1.00 (0,19–2,31)	0.64 (0,20–2,52)	0.82 (0,78–0,86)
**IL-6**	0.91 (0,49–1,42)	2.28* (0,41–8,03)	8.62* † (7,02–9,31)
**IL-1β**	0.87 (0,31–1,79)	1.53 (0,19–2,22)	2.25* (1,86–2,30)
**TGF-β**	1.04 (0,39–1,41)	1.52 (0,28–3,04)	1.80* (1,74–1,95)
**MHC-II**	1.04 (0,64–1,37)	1.15 (0,87–2,00)	1.30 (1,13–1,47)

Median values are shown for each group (n = 3–12) and min-max values are indicated between parentheses. Data were stratified according to the amount of *Pf*Hz in the liver (Li): <70 or >200 nmol *Pf*Hz; Statistically different from PBS administered mice:

* *p*<0.05;

** *p*<0.01;

*** *p*<0.001;

Statistically different from *Pf*Hz Li <70:

† *p*<0.05;

†† *p*<0.01;

FasL, Fas ligand; Hmox1, heme oxygenase 1; ICAM-1, intercellular adhesion molecule-1; IFN-γ, interferon-gamma; IL-, interleukin-; iNOS, inducible nitric oxide synthase; IP-10, interferon gamma inducible protein-10; KC, keratinocyte-derived chemokine; LT-α, lymphotoxin-alpha; MCP-1, monocyte chemotactic protein-1; MHC-II, major histocompatibility complex class II; MMP-9, matrix metalloproteinase-9/gelatinase B; NOX2, NADPH oxidase 2; TGF-β, transforming growth factor-beta; TNF, tumor necrosis factor.

## Discussion

We found no major morphological changes in livers of *P. berghei* or *Pc*AS-infected C57BL/6J mice, nor extensive necrosis, hepatocyte enlargement, steatosis, bile duct damage, or obstruction of blood vessels, sinuses or bile ducts. This is in line with previous observations with *P. berghei* [21], and is consistent with the type of liver injury observed in the majority of patients with hepatic involvement [1],[2]. Mononuclear infiltrations were observed around the central veins and in the parenchyma and were more pronounced in *Pc*AS-infected livers. Furthermore, high amounts of Hz were observed in hepatic macrophages and serum liver enzymes were elevated, especially after infection with *Pc*AS. Because considerable volumes of blood flow through the liver sinusoids and come into close contact with Kupffer cells, circulating particles may be efficiently removed [22]. Upon activation, Kupffer cells may produce inflammatory mediators, e.g. IL-1β, TNF, oxygen radicals and proteases necessary for optimal digestion of the infectious agent upon phagocytosis. Such inflammatory mediators may cause activation of and damage to the surrounding tissue, especially when produced in large quantities. Also blood leukocytes infiltrate the liver and produce inflammatory mediators thereby enhancing hepatic inflammation. A recent paper by Brugat *et al.* confirmed the inflammatory etiology of liver pathology in *Pc*AS-infected C57BL/6 mice, as they found that the increase of serum ALT levels is abrogated in *rag* ^−/−^ and IFN-γ receptor^−/−^ mice [23]. Furthermore, the liver also has an important role in the induction of immune tolerance and it produces high amounts of anti-inflammatory molecules. Deficiencies in immunoregulatory cytokines IL-22 [10] or IL-27 [11], blockade of cytotoxic T lymphocyte antigen 4 (CTLA-4) [12] or deficiency in Hmox1 [13] dramatically increase serum liver enzymes and mortality upon malaria infection, illustrating the importance of immunosuppression by these factors in the liver.

We found high mRNA levels of both pro- and anti-inflammatory mediators in the liver of malaria-infected mice. These levels strongly correlated with hepatic Hz amounts and with serum ALT and AST levels in *Pc*AS-infected mice. Furthermore, intravenous injection of *Pf*Hz into malaria-free C57BL/6J mice induced the expression of a number of these mediators in the liver. The inflammatory effect of *Pf*Hz in the liver corroborates findings obtained with intravenous injections of synthetically produced Hz (sHz, β-hematin) in BALB/c mice [24],[25]. *Pf*Hz and sHz share a similar structure, but differ in size and in the presence of associated proteins and lipids, and sometimes produce opposing effects when used in *in vitro* experiments (reviewed in [26]–[28]). Differences are also found in the size of Hz crystals produced by different parasite species [29], and we found also quantitative differences between the rodent-infecting *P. berghei* and *P. chabaudi* parasites used in this study [19]. However, whether qualitative differences exist between the properties of Hz released by the different species remains to be determined. We used *Pf*Hz that was purified from the supernatant of *in vitro* cultures after it was naturally expelled from rupturing schizonts instead of sHz, since this type of preparation most closely mimics the Hz released during natural infection (reviewed in [26]).

How Hz induces inflammation is not yet fully clarified, but different signaling pathways are activated by Hz *in vitro*. Hz complexes with fibrinogen at their surface are able to trigger the immediate release of reactive oxygen species, TNF and MCP-1 upon Toll-like receptor 4 (TLR4) activation [30], and may thus induce similar inflammatory signaling pathways as bacterial cell wall components like LPS. Also other serum proteins can bind to sHz or to the digestive vacuole containing the Hz, e.g. LPS-binding protein and complement fragments [31],[32], which may further help in Hz recognition and immune activation. Hz also activates the nucleotide-binding oligomerization domain-like receptor pyrin domain containing 3 (NLRP3) inflammasome [33]–[35], whereas contradictory data are found for TLR9 [25],[34],[36]–[39]. More downstream in the signaling cascade, Hz activates several mitogen-activated protein kinases (MAPK) [34],[35],[40]–[43] and nuclear factor kappa-light-chain-enhancer of activated B cells (NF-κB) [41],[42],[44], the latter of which is also activated in peripheral blood mononuclear cells (PBMCs) of patients with uncomplicated malaria [45]. Aside from Hz, also other parasite-derived factors may contribute to the inflammation observed in the liver. For example, malarial glycosylphosphatidylinositols (GPIs) are able to elicit pro-inflammatory responses in monocytes/macrophages [46], and immunization with synthetically produced GPIs diminishes severe pathology in mice [47]. Furthermore, sequestration in the liver may result in localized release of these inflammatory parasite factors [23].

*P. berghei*-infection seems to cause less hepatocyte damage compared to *Pc*AS-infection, although larger amounts of Hz were observed in the livers of *P. berghei*-infected mice regardless of the genetic background of the mouse strain used. The higher hepatic Hz levels can be explained by the higher Hz production levels of *P. berghei* parasites compared to *Pc*AS parasites [19]. A possible explanation for the lower amount of hepatocyte damage observed with *P. berghei* is that inflammatory responses might be suppressed in the liver with *P. berghei*, as it was demonstrated that large amounts of Hz may impair specific immune functions [48]–[52]. We found a strong negative correlation between hepatic Hz levels and the expression of MHC class II on monocytes/macrophages, as was also found *in vitro* with human monocytes [53] and murine dendritic cells [50], indicating that antigen presentation and subsequent T cell responses may be impaired and inflammatory responses in the liver may be limited by Hz. Furthermore, no correlations were found between Hz, pathology and inflammation in the liver of *P. berghei*-infected mice, which is in striking contrast with the strong correlations found between pulmonary Hz levels, MA-ARDS disease parameters and pulmonary inflammation [19]. This might be due to the differential amounts of Hz found in both organs, since Hz levels are about 25-fold higher in livers than in lungs [3]. Urban and Todryk suggested that the activating *versus* toxic effects of Hz on the immune system may depend on the timing and the amount of Hz ingested, with low amounts of Hz early during infection leading to activation and high amounts of Hz with increasing parasitemia leading to inactivation [52]. This biphasic response of the immune system on differential amounts of Hz might also occur in our model in which low to medium levels of Hz as found in e.g. lungs of *P. berghei*-infected mice and livers of *Pc*AS-infected mice correlated with inflammation, whereas high Hz levels as e.g. in livers of *P. berghei*-infected mice appeared to suppress the immune response. On the other hand, the high inflammatory environment in livers of *Pc*AS-infected mice might also indicate that parasite killing takes place in the liver as was already suggested by Good *et al.* [54] and Playfair *et al.* [55]. Even though we did not observe parasites in the liver by histologic analysis, hepatic sequestration was observed by Brugat *et al.* [23]. This discrepancy may be due to time differences when the organs were harvested.

As a conclusion, *Pc*AS induces liver pathology in mice to a similar degree as *P. falciparum* in many patients. In *Pc*AS-infected C57BL/6J mice striking correlations were established between Hz levels, liver pathology, and the expression of inflammatory mediators. The causative role of Hz was evidenced by intravenous injection of *Pf*Hz indicating that Hz activates the transcription of inflammation-inducible genes in the liver. As a result several chemokines, cytokines and proteases are secreted that may contribute to hepatocyte damage and subsequent leakage of liver enzymes into the circulation. In livers of *P. berghei*-infected mice no correlations with inflammatory gene expression were documented despite higher levels of Hz compared to livers from *Pc*AS-infected mice.

## Supporting Information

Figure S1 **Liver pathology depends on the parasite strain and is only marginally influenced by host genetics.** C57BL/6J and BALB/c mice were infected with *Pb*NK65 or *Pc*AS and sacrificed at different time points after infection. As C57BL/6J mice infected with *Pb*NK65 die shortly after day 10, no data were available for this group at day 13. (A, B) Peripheral parasitemia levels. Means are indicated ± SEM (n = 8–10 for each group). (C, D) Weights of perfused livers. Means are indicated ± SEM (n = 8–10 for each dot). (E–H) Serum levels of ALT (panels E, F) and AST (panels G, H). Please notice the almost 10-fold difference in ALT and AST levels on the Y-axis for *Pb*NK65 and *Pc*AS infections. (I–J) Quantity of hemozoin (Hz)/mg liver tissue at different time points after infection with *Pb*NK65 (I) or *Pc*AS (J). Data are pooled from two separate experiments with similar results. Each dot represents the results from an individual mouse. Horizontal bars between individual data points represent group medians and horizontal lines with asterisks on top indicate statistical differences between groups. * *p*<0.05, ** *p*<0.01 and *** *p*<0.001.Figure S2. Hz in marginating cells but not in endothelial cells.
(DOC)

Figure S2 **Hz in marginating cells but not in endothelial cells.** Higher magnification of liver endothelium and marginating cells containing hemozoin (Hz, brown crystals) in hematoxylin-eosin stained paraffin sections of livers from C57BL/6J mice infected with *Pb*ANKA (day 8), *Pb*NK65 (day 10) or *Pc*AS (day 10).
(DOC)

Figure S3 **Immunohistochemistry for Gr-1 in mouse liver after** ***PcAS*** **-infection.** Paraffin-embedded sections were prepared from the liver of a mouse mouse infected with *Pc*AS (day 10), and stained for Gr-1. White arrow, Gr-1^hi^ monocyte; black arrow, Gr-1^+^ macrophage containing Hz (brown granules). Representative images are shown (original magnification, 400× and 1000×; scale bars, 50 µm).
(DOC)
