# Supplementary material for: Hemozoin Induces Hepatic Inflammation in Mice and Is Differentially Associated with Liver Pathology Depending on the Plasmodium Strain
Source: PLoS One. 2014 Nov 24;9(11):e113519. doi: 10.1371/journal.pone.0113519 (PMC4242621; doi:10.1371/journal.pone.0113519)
Supplement: Figure S1 — Liver pathology depends on the parasite strain and is only marginally influenced by host genetics. C57BL/6J and BALB/c mice were infected with PbNK65 or PcAS and sacrificed at different time points after infection. As C57BL/6J mice infected with PbNK65 die shortly after day 10, no data were available for this group at day 13. (A, B) Peripheral parasitemia levels. Means are indicated ± SEM (n = 8–10 for each group). (C, D) Weights of perfused livers. Means are indicated ± SEM (n = 8–10 for each dot). (E–H) Serum levels of ALT (panels E, F) and AST (panels G, H). Please notice the almost 10-fold difference in ALT and AST levels on the Y-axis for PbNK65 and PcAS infections. (I–J) Quantity of hemozoin (Hz)/mg liver tissue at different time points after infection with PbNK65 (I) or PcAS (J). Data are pooled from two separate experiments with similar results. Each dot represents the results from an individual mouse. Horizontal bars between individual data points represent group medians and horizontal lines with asterisks on top indicate statistical differences between groups. * p<0.05, ** p<0.01 and *** p<0.001.Figure S2. Hz in marginating cells but not in endothelial cells. (DOC) [file pone.0113519.s001.doc]

**
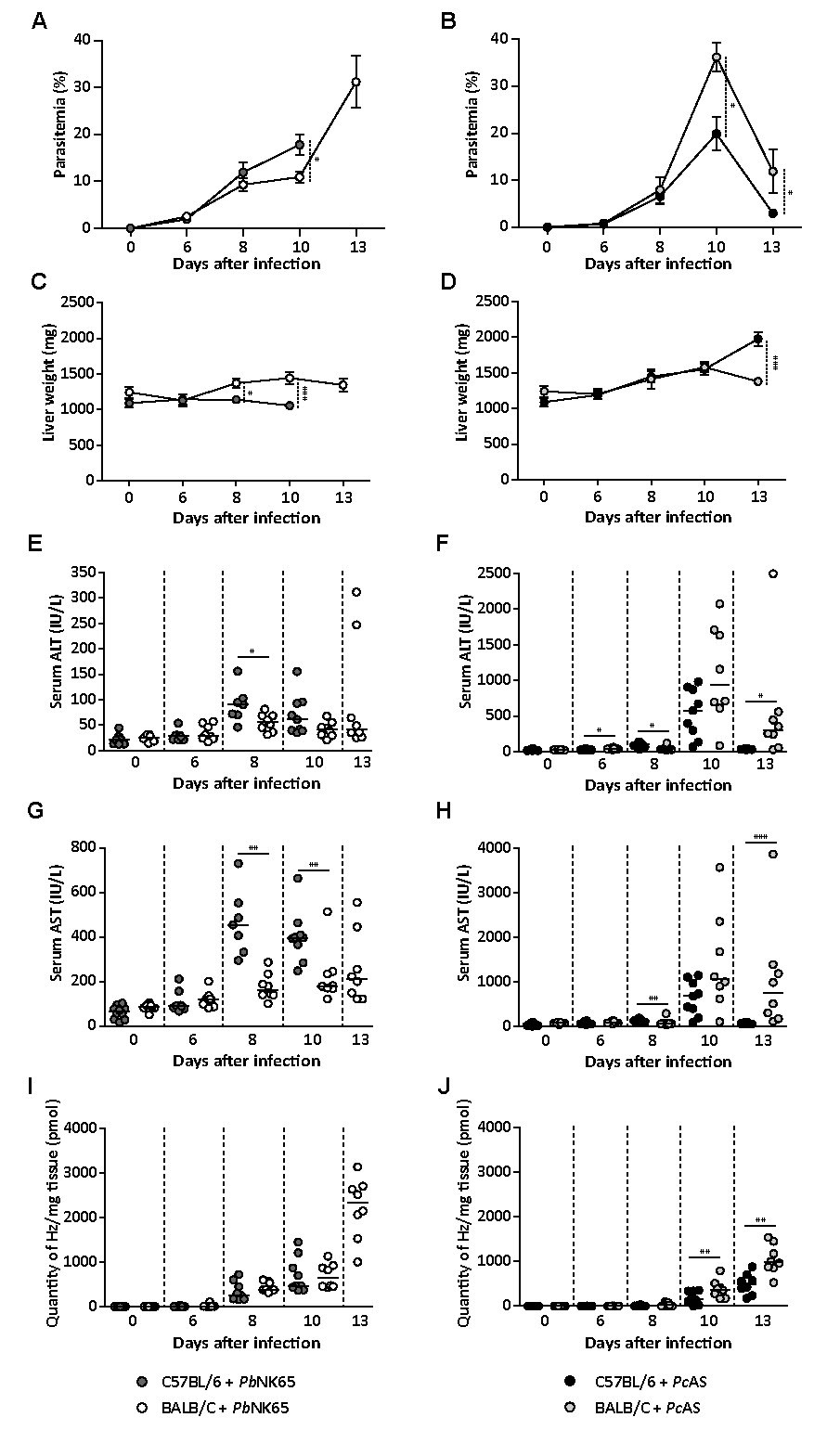
**

**Fig S1. Liver pathology depends on the parasite strain and is only marginally influenced by host genetics.** C57BL/6J and BALB/c mice were infected with *Pb*NK65 or *Pc*AS and sacrificed at different time points after infection. As C57BL/6J mice infected with *Pb*NK65 die shortly after day 10, no data were available for this group at day 13. (A, B) Peripheral parasitemia levels. Means are indicated ± SEM (n = 8 – 10 for each group). (C, D) Weights of perfused livers. Means are indicated ± SEM (n = 8 – 10 for each dot). (E-H) Serum levels of ALT (panels E, F) and AST (panels G, H). Please notice the almost 10-fold difference in ALT and AST levels on the Y-axis for *Pb*NK65 and *Pc*AS infections. (I-J) Quantity of hemozoin (Hz)/mg liver tissue at different time points after infection with *Pb*NK65 (I) or *Pc*AS (J). Data are pooled from two separate experiments with similar results. Each dot represents the results from an individual mouse. Horizontal bars between individual data points represent group medians and horizontal lines with asterisks on top indicate statistical differences between groups. * *p* < 0.05, ** *p* < 0.01 and *** *p* < 0.001.
