# Supplementary material for: Hemozoin Induces Hepatic Inflammation in Mice and Is Differentially Associated with Liver Pathology Depending on the Plasmodium Strain
Source: PLoS One. 2014 Nov 24;9(11):e113519. doi: 10.1371/journal.pone.0113519 (PMC4242621; doi:10.1371/journal.pone.0113519)
Supplement: Figure S2 — Hz in marginating cells but not in endothelial cells. Higher magnification of liver endothelium and marginating cells containing hemozoin (Hz, brown crystals) in hematoxylin-eosin stained paraffin sections of livers from C57BL/6J mice infected with PbANKA (day 8), PbNK65 (day 10) or PcAS (day 10). (DOC) [file pone.0113519.s002.doc]

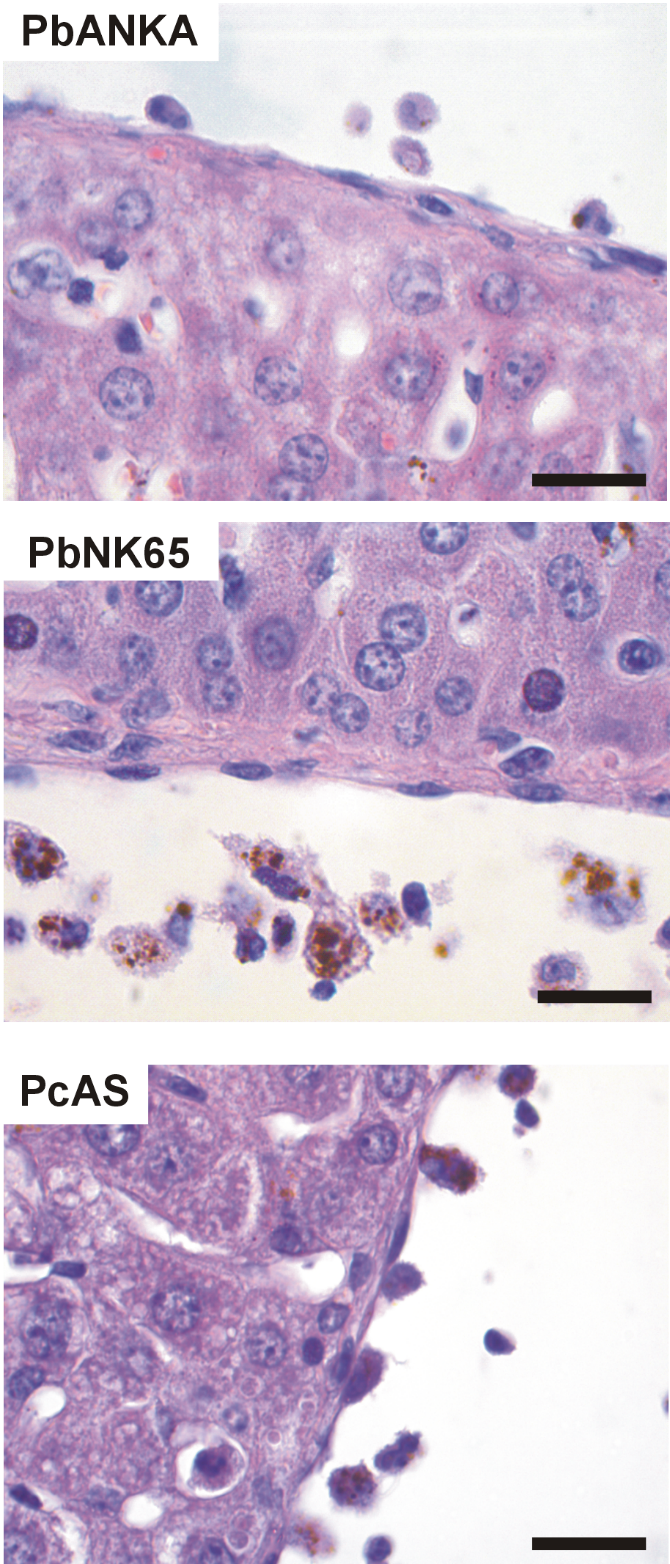


**Figure S2. Hz in marginating cells but not in endothelial cells.**

Higher magnification of liver endothelium and marginating cells containing hemozoin (Hz, brown crystals) in hematoxylin-eosin stained paraffin sections of livers from C57BL/6J mice infected with *Pb*ANKA (day 8), *Pb*NK65 (day 10) or *Pc*AS (day 10).
