# Supplementary material for: Hemozoin Induces Hepatic Inflammation in Mice and Is Differentially Associated with Liver Pathology Depending on the Plasmodium Strain
Source: PLoS One. 2014 Nov 24;9(11):e113519. doi: 10.1371/journal.pone.0113519 (PMC4242621; doi:10.1371/journal.pone.0113519)
Supplement: Figure S3 — Immunohistochemistry for Gr-1 in mouse liver after PcAS -infection. Paraffin-embedded sections were prepared from the liver of a mouse mouse infected with PcAS (day 10), and stained for Gr-1. White arrow, Gr-1hi monocyte; black arrow, Gr-1+ macrophage containing Hz (brown granules). Representative images are shown (original magnification, 400× and 1000×; scale bars, 50 µm). (DOC) [file pone.0113519.s003.doc]

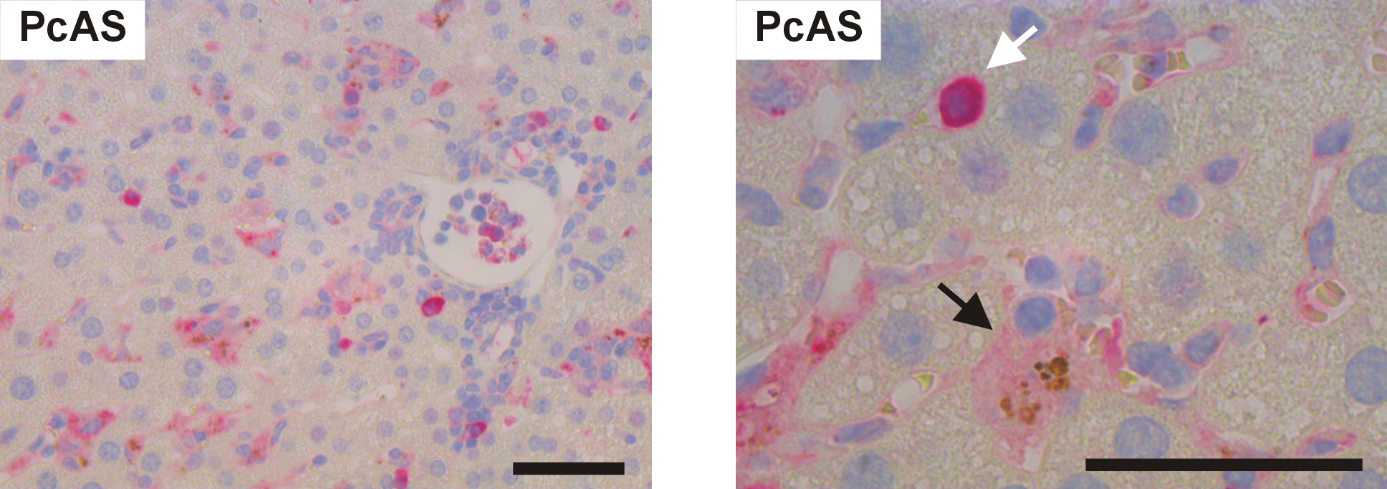


**Figure S3. Immunohistochemistry for Gr-1 in mouse liver after *PcAS*-infection.**

Paraffin-embedded sections were prepared from the liver of a mouse mouse infected with *Pc*AS (day 10), and stained for Gr-1. White arrow, Gr-1^hi^ monocyte; black arrow, Gr-1^+^ macrophage containing Hz (brown granules). Representative images are shown (original magnification, 400x and 1000x; scale bars, 50 µm).
